# High-physiological and supra-physiological 1,2-^13^C_2_ glucose focal supplementation to the traumatised human brain

**DOI:** 10.1177/0271678X231173584

**Published:** 2023-05-08

**Authors:** Matthew G Stovell, Duncan J Howe, Eric P Thelin, Ibrahim Jalloh, Adel Helmy, Mathew R Guilfoyle, Peter Grice, Andrew Mason, Susan Giorgi-Coll, Clare N Gallagher, Michael P Murphy, David K Menon, T Adrian Carpenter, Peter J Hutchinson, Keri LH Carpenter

**Affiliations:** 1 Division of Neurosurgery, Department of Clinical Neurosciences, University of Cambridge, Cambridge, UK; 2 Department of Neurosurgery, The Walton Centre, Liverpool, UK; 3 Department of Chemistry, University of Cambridge, Cambridge, UK; 4 Department of Clinical Neuroscience, Karolinska Institutet, Stockholm, Sweden; 5 Department of Neurology, Karolinska University Hospital, Stockholm, Sweden; 6 Division of Neurosurgery, Department of Clinical Neurosciences, University of Calgary, Calgary, Canada; 7 MRC Mitochondrial Biology Unit, University of Cambridge, Cambridge, UK; 8 Division of Anaesthesia, Department of Medicine, University of Cambridge, Cambridge, UK; 9 Wolfson Brain Imaging Centre, Department of Clinical Neurosciences, University of Cambridge, Cambridge, UK

**Keywords:** Brain metabolism, 1,2-^13^C_2_ glucose, microdialysis, NMR, traumatic brain injury (human)

## Abstract

How to optimise glucose metabolism in the traumatised human brain remains unclear, including whether injured brain can metabolise additional glucose when supplied. We studied the effect of microdialysis-delivered 1,2-^13^C_2_ glucose at 4 and 8 mmol/L on brain extracellular chemistry using bedside ISCUS*flex*, and the fate of the ^13^C label in the 8 mmol/L group using high-resolution NMR of recovered microdialysates, in 20 patients. Compared with unsupplemented perfusion, 4 mmol/L glucose increased extracellular concentrations of pyruvate (17%, p = 0.04) and lactate (19%, p = 0.01), with a small increase in lactate/pyruvate ratio (5%, p = 0.007). Perfusion with 8 mmol/L glucose did not significantly influence extracellular chemistry measured with ISCUS*flex*, compared to unsupplemented perfusion. These extracellular chemistry changes appeared influenced by the underlying metabolic states of patients’ traumatised brains, and the presence of relative neuroglycopaenia. Despite abundant ^13^C glucose supplementation, NMR revealed only 16.7% ^13^C enrichment of recovered extracellular lactate; the majority being glycolytic in origin. Furthermore, no ^13^C enrichment of TCA cycle-derived extracellular glutamine was detected. These findings indicate that a large proportion of extracellular lactate does not originate from local glucose metabolism, and taken together with our earlier studies, suggest that extracellular lactate is an important transitional step in the brain’s production of glutamine.

## Introduction

The leading cause of death and disability in young adults in the developed world is traumatic brain injury (TBI). Initial survivors of severe TBI may subsequently suffer further brain damage through secondary insults including dysfunction of brain energy metabolism, despite the best current clinical management, affecting patient outcome.^1^ While various brain metabolic fuels are reported, including lactate,^2–5^ glucose is conventionally regarded as the primary substrate for brain energy metabolism.^6–8^

After TBI, changes in brain glucose metabolism have been demonstrated with typically lower cerebral microdialysate concentrations of glucose (e.g. 1.1 mmol/L)^9^ than non-TBI control patients (1.7–2.0 mmol/L).^10,11^ It is unclear whether low microdialysis glucose within TBI cohorts is pathological or adaptive. Furthermore, extremes of both high and low microdialysis glucose are associated with worse outcomes.^8,9^ Low microdialysate glucose (neuroglycopenia) may result from reduced glucose transport across the blood brain barrier, or greater cellular uptake of glucose from the extracellular space, or stem from ischemia. The latter is less likely with modern neuro-intensive care protocols targeting partial brain oxygen pressure (PbtO_2_) and cerebral perfusion pressure (CPP) thresholds, but the traumatised brain may have higher serum glucose requirements than the uninjured brain. Inadequate cellular uptake may explain high microdialysate glucose associated with unfavourable outcome in a large study of TBI patients.^9^ Studies comparing ‘tight’ and ‘loose’ control of serum glucose reported that tight serum glucose control was associated with more frequent episodes of critically low microdialysis glucose,^12–14^ so more permissive serum glucose control is often advocated.

Cerebral glucose metabolism changes after TBI are thus complex. The injured brain appears more dependent on serum glucose to maintain cerebral glucose, and since both high and low brain glucose levels are statistically associated with unfavourable outcome, an optimal range seems likely. There are currently insufficient data to define this,^8^ and no existing evidence whether supplementing the traumatised brain with additional glucose will support its metabolism and be beneficial,^8^ as while serum glucose and glycaemic control influence brain glucose, the relationship may be altered in injured brain.^8^

We aimed to determine if the traumatised human brain could metabolise supplementary glucose when delivered focally into the brain extracellular fluid. We expected additional glucose metabolism to be expressed as an increase in its metabolites extracellularly: pyruvate and lactate in the recovered microdialysates.^15^ 1,2-^13^C_2_ glucose was perfused via a cerebral microdialysis catheter at a ‘high-physiological’ concentration (4 mmol/L), and a ‘supra-physiological’ concentration (8 mmol/L) to study any dose response to this focal supplementation, thereby avoiding systemic metabolism and the blood brain barrier. We used ^13^C high-resolution nuclear magnetic resonance (NMR) analysis of the recovered microdialysates, expecting to detect ^13^C lactate and ^13^C glutamine as these glucose metabolites are typically sustained and relatively abundant extracellularly in brain.^3,16,17^

## Materials and methods

### Patients

We recruited patients aged >16 years with a severe/moderate cranial trauma (post-resuscitation Glasgow Coma Scale (GCS) ≤ 12), who additionally required sedation and mechanical ventilation for intracranial hypertension and/or airway protection. Patients were treated using our standard TBI management protocols including endotracheal intubation, ventilation, sedation, neuromuscular blockade and maintenance of serum glucose concentration within the target range 4–10 mmol/L.^18^ Informed written assent was obtained from patients’ relatives. The study was conducted in conformation with the spirit and the letter of the Declaration of Helsinki. The National Research Ethics Service, Committee East of England–Cambridge Central (REC Reference No.11/EE/0463) approved the protocol. Data from four of the patients who received high-physiological (4 mmol/L) 1,2-^13^C_2_ glucose by microdialysis were from a previous study.^11^ The others were not in any previous ^13^C studies.

### Perfusion fluid and ^13^C-labelled substrate

CNS Perfusion Fluid (M Dialysis AB, Stockholm, Sweden) consisted of NaCl (147 mmol/L), KCl (2.7 mmol/L), CaCl_2_ (1.2 mmol/L), and MgCl_2_ (0.85 mmol/L) in water. 1,2-^13^C_2_ glucose (isotopic enrichment 99%, chemical purity 99%) from Cambridge Isotope Laboratories (Tewksbury, MA, USA) was formulated at 4 or 8 mmol/L in CNS perfusion fluid by the Pharmacy Manufacturing Unit, Ipswich Hospital NHS Trust (Ipswich, UK) who tested the formulations to verify purity, sterility, freedom from endotoxins and absence of pyrogenicity, compliant with current regulations.

### Microdialysis technique

CMA 71 microdialysis catheters (membrane 10 mm, cut-off 100 kDa, M Dialysis AB) were directed into normal-appearing brain (white matter); neither into nor adjacent to CT-visible lesions, per the 2014 Consensus Statement guidelines.^8^ The microdialysis catheter was placed either via a craniotomy, or through a triple-lumen cranial access device (Technicam, Newton Abbot, UK) together with an intracranial pressure monitor (Codman, Raynham, MA, USA) and a Licox PbtO_2_ sensor (GMS, Kiel-Mielkendorf, Germany) when available. Catheters were perfused at 0.3 µL/min with plain, unsupplemented CNS Perfusion Fluid for at least 24 h. The first hour of microdialysate collected was never used for clinical monitoring (to eliminate any artefacts from insertion trauma and the pump flush sequence). Then, for a period of 24 h, the perfusion fluid was changed to CNS perfusion fluid supplemented with either 4 mmol/L or 8 mmol/L 1,2-^13^C_2_ glucose. Afterwards, the perfusion fluid was reverted to plain unsupplemented CNS perfusion fluid. Microdialysate collection vials were changed hourly and analysed on a bedside ISCUS*flex* analyser (M Dialysis AB) for glucose, lactate, pyruvate, glycerol and glutamate. Microdialysates were frozen at -80°C if storage >24 h was necessary before pooling for subsequent NMR analysis.

### High resolution NMR acquisition and analysis

After bedside ISCUS*flex* analysis, brain microdialysate samples from patients perfused with 8 mmol/L 1,2-^13^C_2_ glucose were collected and pooled into 24-hour supplementation periods for each patient. Samples from patients perfused with 4 mmol/L 1,2-^13^C_2_ glucose were not analysed with ^13^C NMR due to limited availability of this resource and the existence of our similar previous studies.^11^ Each patient’s pooled sample (180 µL) was added to 20 µL of deuterium oxide (D_2_O) and 50 µL of 24 mmol/L 2,2-dimethyl-2-silapentane-5-sulfonate sodium salt (DSS) internal reference standard (Sigma-Aldrich, Poole, Dorset, UK). NMR analysis was performed using 3 mm NMR tubes (Hilgenberg GmbH, Malsfeld, Germany) in a Bruker Avance III HD 500 MHz spectrometer (Bruker BioSpin GmbH, Karlsruhe, Germany) using a dual ^1^H/^13^C cryoprobe (CP DUL500C/H, Bruker BioSpin GmbH) for ^13^C and ^1^H spectral acquisition (for further details, see Supplementary Information online). Peaks were identified by reference to our own standards and online NMR databases (BMRB—Biological Magnetic Resonance Bank, University of Wisconsin^19^ and HMDB—Human Metabolome Database, Genome Alberta^20^) Chemical shifts were expressed in ppm (Hz per MHz) referenced to DSS at zero ppm. Peak areas for DSS and lactate signals were integrated using TopSpin.

To quantify the lactate signals in the ^13^C NMR spectra, calibration was performed with a series of known concentrations of standard lactate,^11^ in CNS perfusion fluid and added to NMR tubes at the same volumes as for microdialysates, with the same fixed concentrations and volumes of D_2_O and DSS as an internal standard as used for the microdialysates samples and run under identical NMR conditions. To quantify the microdialysate spectra, peak areas relative to the DSS internal standard were used with reference to calibration curves derived from the lactate standards, showing a linear relationship between peak areas (ratio to DSS internal standard) and concentrations.

Fractional enrichment (%) is defined as 100×[^13^C]/([^13^C]+[^12^C]) where square brackets indicate concentrations of the relevant species. [^13^C] was determined from the calibrated ^13^C NMR spectra (above). [^12^C] was determined from the ^1^H spectra, measuring the peak areas of lactate C3 methyl protons that give an ^1^H NMR doublet at 1.32 ppm (from protons attached to ^12^C), relative to the peak area of DSS internal standard.

^13^C natural abundance is 1.1% of all carbon atoms, and ^13^C results for the lactate ^13^C singlet signals were expressed after subtracting this natural background. ^13^C doublet signals were not background-subtracted because the probability of two ^13^C atoms occurring next to each other naturally is 0.01% (=1.1% × 1.1%).

The pentose phosphate pathway (PPP)-derived 3-^13^C lactate ratio to glycolysis-derived 2,3-^13^C_2_ lactate was expressed in the form 1:N where N is the concentration of lactate derived from the ^13^C doublet at 22.8, divided by the concentration of lactate represented by the ^13^C singlet at 22.8 ppm (after first subtracting the singlet’s ^13^C natural abundance background).

### Statistical analysis

Statistical data analysis utilised R (version 3.5.1, www.r-project.org). Demographics of patients who received 4 mmol/L and 8 mmol/L glucose were compared using Mann-Whitney U test. For ISCUS*flex* results, changes between baseline and glucose supplementation periods were excluded for 2 h after changeover from pre-supplementation to glucose supplementation, and changeover from glucose supplementation to post-supplementation, to allow for ‘run-in’ and ‘washout’.^17^ Each patient’s pre- and post-supplementation data were combined as a baseline period for that individual, thereby allowing for underlying trends in patient physiology. Heterogeneity (frequent in TBI cohorts) was accounted for by a linear mixed effects model (‘*lmer*’ in R package *lme4*^21^) that allows for clustering of data, and has a different random effect for each patient. Also, Wilcoxon signed rank test was used to corroborate statistical results. Only those patients with microdialysates from *both* pre- and post-supplementation baseline periods plus a supplementation data period were included in the ISCUS*flex* data analysis. The Shapiro-Wilk normality test was also performed. Our detailed data analysis of ISCUS*flex* results focussed mainly on microdialysis glucose, lactate, pyruvate and lactate/pyruvate ratio (LPR), as our primary question was whether the traumatised brain could metabolise additional glucose, and interpretation of microdialysate glycerol is ambiguous (as it can be a glucose metabolite or a breakdown product of lipid),^8^ and several patients had incomplete glutamate microdialysis data.

## Results

### Patients and monitoring

Twenty TBI patients (14 M, 6 F) were recruited; mean age 39 y (median 33 y, range 16 y–65 y), and post-resuscitation mean GCS 6. Injury causes were mostly road traffic collisions and falls. The commonest surgical diagnoses were cerebral contusions and acute subdural haematomas. For detailed demography, see Supplementary Table 1. There were no complications from insertion of microdialysis catheters, nor related to the perfusion of study substrate; specifically, no cerebral haematomas or contusions were noted on CT imaging after catheter insertion. Throughout the study period, adequate CPP was maintained in all patients (>60 mmHg) and no patients suffered from intractable raised ICP (>25 mmHg). PbtO_2_ data was available for 7/20 patients (mean PbtO_2_ 27 mmHg). No patients suffered from cerebral ischaemia (PbtO_2_<15 mmHg).

### Patient baseline characteristics

There were no significant differences (Mann-Whitney U, *p* *>* *0.1*) between the two groups of patients – those who received 4 mmol/L or 8 mmol/L glucose – for baseline GCS, Extended Glasgow Outcome Scale (GOS-E),^22^ time interval between injury and period of supplementation (mean 2.9 days), nor serum glucose or serum lactate during the 1,2-^13^C_2_ glucose supplementation period. However, there was a statistically significant difference (*p* *<* *0.0001, lmer*) between the two patient groups for microdialysate glucose, lactate and pyruvate at baseline. For ’baseline’ definition, see *Statistical Analysis* above. Baseline glucose differences between the two groups of patients are likely due to inherent heterogeneity between TBI patients, and our cohort being small. Microdialysate baseline glucose was 32% higher in the 8 mmol/L group versus the 4 mmol/L group, while lactate and pyruvate were 21% and 13% lower respectively, although the baseline lactate/pyruvate ratio (LPR) was only 4% lower in the 8 mmol/L than in the 4 mmol/L group (Table 1, Supplementary Table 2).

**Table 1.** ISCUS*flex* results for microdialysates at baseline and during microdialysis supplementation with 1,2-^13^C_2_ glucose.

ISCUSflex analyte	Supp. conc. (mmol/L)	Baseline Mean (sd)	Supp. Mean (sd)	% change mean	*lmer p*	Baseline Median (IQR)	Supp. Median (IQR)	% change median	*Wilcoxon p*
Glucose (mmol/L)	4	1.22 (0.70)	4.07 (0.80)	↑233%	*0.0001*	1.05 (0.79)	3.77 (0.96)	↑259%	*0.001*
	8	1.61 (1.38)	7.56 (0.91)	↑309%	*0.0001*	0.92 (1.14)	7.47 (1.13)	↑708%	*0.008*
Lactate (mmol/L)	4	3.26 (1.36)	3.89 (1.92)	↑19%	*0.0001*	3.07 (1.28)	3.71 (1.91)	↑21%	*0.01*
	8	2.56 (1.25)	2.50 (0.93)	↓2%	*0.17*	2.18 (0.52)	2.16 (0.30)	↓1%	*0.8*
Pyruvate (µmol/L)	4	113 (41)	132 (52)	↑17%	*0.0001*	106 (35)	122 (72)	↑15%	*0.04*
	8	98 (37)	95 (31)	↓2%	*0.12*	78 (33)	86 (40)	↑10%	*0.64*
LPR	4	27.8 (9.2)	29.3 (9.8)	↑5%	*0.002*	23.6 (12.0)	26.4 (12.2)	↑12%	*0.007*
	8	26.7 (6.7)	27.1 (6.1)	↑1.5%	*0.17*	26.0 (3.4)	27.6 (4.8)	↑6%	*0.33*
Glycerol (µmol/L)	4	109 (76)	118 (85)	↑8%	*0.0001*	110 (79)	88 (82)	↓20%	*0.2*
	8	93 (37)	107 (55)	↑15%	*0.0001*	85 (36)	101 (36)	↑19%	*0.2*
Glutamate(µmol/L)	4	3.24 (3.37)	8.06 (8.11)	↑149%	*0.3*	2.35 (2.35)	6.75 (4.27)	↑187%	*0.9*
	8	15.30 (34.10)	12.10 (29.50)	↓21%	*0.0001*	3.89 (3.68)	1.90 (1.06)	↓51%	*0.016*

Group means (with sd) and medians (with IQR) from periods of baseline (pre- and post- supplementation) and during supplementation with 1,2-^13^C_2_ glucose (Supp.). Total number (n) of patients whose data were included in this table was 18. One of these 18 patients had two catheters, so the results here in Table 1 are from 19 catheters. For further details, see Supplemental Table 1 (patient demographics). Significance was determined using R by linear mixed effects model (lmer) of patient individual hourly data and Wilcoxon signed rank of patient averaged data. Test for normality of metabolite data was performed in R using Shapiro-Wilk test. Patient hourly data were not normally distributed. Although the majority of patient-averaged data was found to be normally distributed, Wilcoxon signed rank test was used due to the small number of observations and the inclusion of analytes with non-normally distributed data. Up- and down- arrows denote % change increases and decreases respectively. sd; standard deviation; IQR; interquartile range.

### Evidence from ^13^C labelling patterns

**Figure 1.**
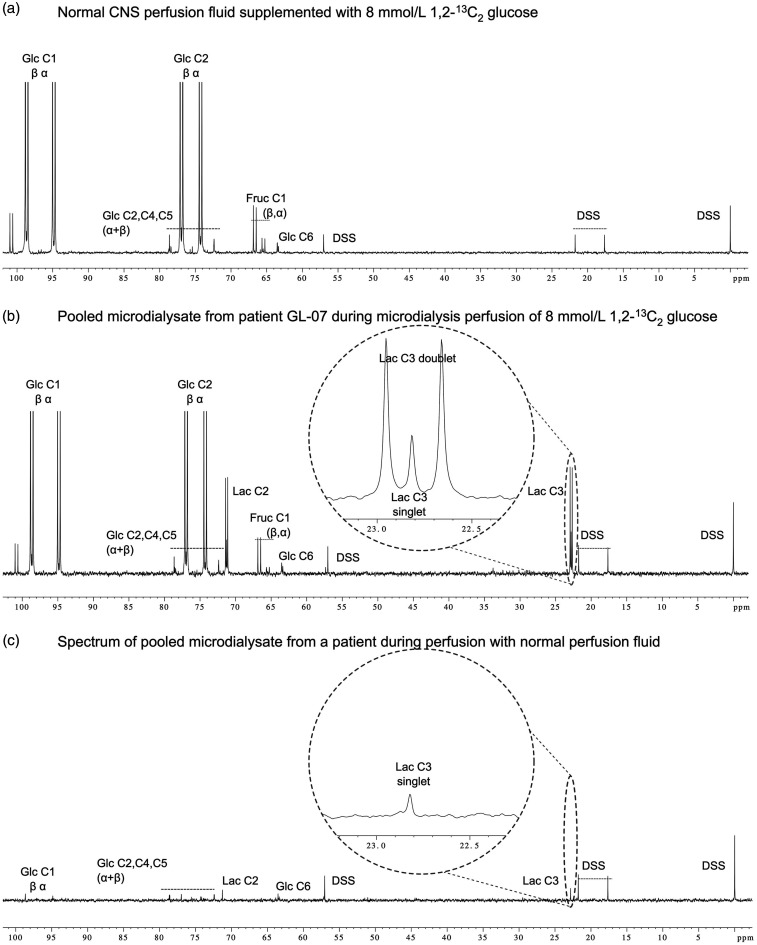
Example ^13^C NMR spectra: *Panel a* CNS perfusion fluid supplemented with 8 mmol/L 1,2-^13^C_2_ glucose, prior to infusion. The large α and β peaks for ^13^C at glucose position C1 and C2 can be seen dominating the spectrum, with small peaks representing natural abundance of ^13^C at the unenriched positions within the molecule. *Panel b* pooled microdialysate from a patient during their 24 h of 8 mmol/L 1,2-^13^C_2_ glucose supplementation. Lactate can be seen at 22.8 ppm (C3) and 71.2 ppm (C2). The C3 peak is expanded, showing in greater detail the ^13^C labelled doublet and the small ^13^C singlet inside the doublet. The doublet represents glycolytic metabolism of supplemented 1,2-^13^C_2_ glucose, and the singlet a combination of pentose phosphate pathway-metabolised 1,2-^13^C_2_ glucose supplement and the natural abundance of ^13^C label in endogenous lactate. This singlet within a doublet was visible in all patients’ microdialysates analysed with high resolution NMR. *Panel c* pooled microdialysate from a patient during a period of ‘baseline’ perfusion with unsupplemented perfusion fluid. Small singlet peaks can be seen corresponding to lactate and glucose, representing the natural abundance background of ^13^C in these molecules. All spectra were analysed with TopSpin (Bruker GmbH), referenced to internal standard DSS. Small peaks for ^13^C-labelled fructose are visible in Panel a and Panel b, representing a small proportion of ^13^C-labelled glucose that has undergone isomerisation which occurs spontaneously in solution. Glc: Glucose; Fruc: fructose; DSS: 4,4-dimethyl-4-silapentane-1-sulfonate sodium salt internal reference standard; Lac: lactate.

**Figure 2.**
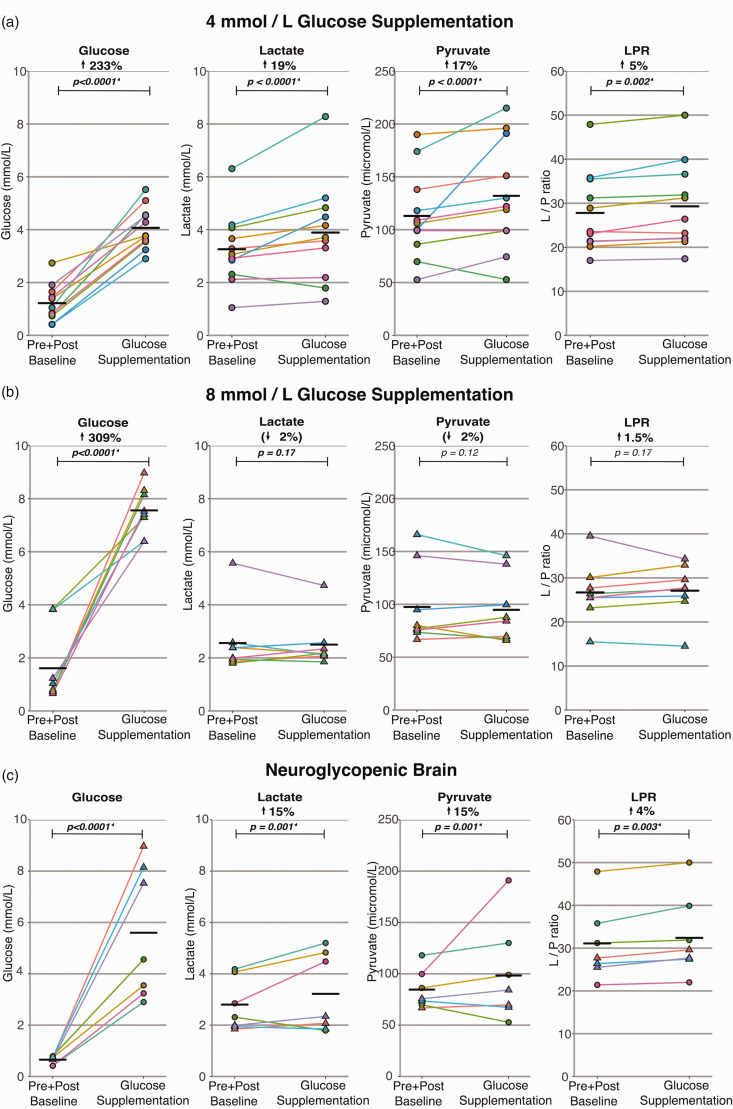
ISCUS*flex* bedside analyser measurements showing the effect of 1,2-^13^C_2_ glucose supplementation via microdialysis on brain extracellular chemistry. Each pair of data-points indicates mean levels at baseline and during 1,2-^13^C_2_ glucose perfusion, respectively, for that patient. Supplementation was for ≈ 24 h. Baseline denotes ≈ 24 h of perfusion with plain (unsupplemented) CNS-perfusion fluid; the baseline value represents combined data pre- and post-supplementation (to account for any underlying trends). Upper 4 *Panel a* (circles) denote results from patients supplemented with 1,2-^13^C_2_ glucose at 4 mmol/L, middle 4 *Panel b* (triangles) denote results from patients supplemented with 1,2-^13^C_2_ glucose at 8 mmol/L, lower 4 *Panel c* (circles and triangles) denote results from subset of these patients from both groups with low baseline cerebral glucose (<0.8 mmol/L), black crossbars on graphs denote averages (means) of individual patient means. P-values calculated using linear mixed effects model in R. LPR: lactate/pyruvate ratio. Note that in Panel a the LPR data points for two of the patients who received 4 mM 1,2-^13^C_2_ glucose almost exactly coincide (see Supplemental Table 2 for patients GL-17 and GL-19).

**Figure 3.**
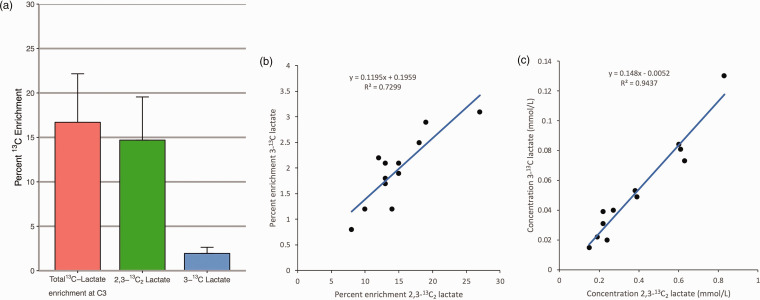
Bar chart of mean percentage ^13^C enrichment in lactate carbon position 3 after supplementation with 8 mmol/L 1,2-^13^C_2_ glucose, and correlation between glycolysis and PPP derived lactate. Panel a: Error bars denote standard deviation. ^1^H and ^13^C high-resolution NMR analysed with TopSpin. ‘Total 3-^13^C Lactate enrichment at C3’ denotes total fractional (percentage) ^13^C enrichment at the C3 position of lactate (the methyl group), which represents glycolytic and pentose phosphate pathway (PPP) metabolism of supplemented glucose. ‘2,3-^13^C_2_ Lactate’ denotes percent of all lactate that is doubly-labelled (2,3-^13^C_2_ lactate doublet), representing glycolytic-derived lactate. ‘3-^13^C Lactate (endog. subtracted)’ denotes percent of all lactate that is singly labelled (3-^13^C lactate singlet after subtraction of the natural abundance background ^13^C) produced via the PPP. There was a strong linear correlation between glycolysis-derived lactate (2,3-^13^C_2_ lactate) and pentose phosphate pathway (PPP)-derived lactate (3-^13^C lactate, excluding natural abundance) with a y-intercept of close to zero in these patients who received 8 mmol/L 1,2-^13^C_2_ glucose, suggesting that these two metabolic pathways are regulated in unison in TBI. Relationships expressed as fractional (percentage) ^13^C enrichment (*Panel b)* and ^13^C concentration (mmol/L) (*Panel c).* Each data-point represents 24 x 1 h pooled microdialysis vials from each individual patient. Numerical values are in Table 2. Panel b: 3-^13^C lactate fractional (percentage) enrichment (*y*-axis), plotted versus 2,3-^13^C_2_ lactate fractional (percentage) enrichment (*x*-axis). Linear relationship, *y = *0.1195*x* + 0.1959, Pearson’s r^2^ = 0.7299. Panel c: 3-^13^C lactate concentration in mmol/L (*y*-axis), plotted versus 2,3-^13^C_2_ lactate concentration in mmol/L (*x*-axis). Linear relationship, *y = *0.148*x* - 0.0052, Pearson’s r^2^ = 0.9437. Thus, approximately 1 molecule of 3-^13^C lactate is produced by the PPP for every 6.8 molecules of 2,3-^13^C lactate produced by glycolysis, when 1,2-^13^C_2_ glucose is metabolised.

The appearance of doubly-labelled lactate (2,3-^13^C_2_ lactate) in the recovered microdialysates showed unequivocally that the brain metabolised by glycolysis the exogenous 1,2-^13^C_2_ glucose delivered via the microdialysis catheter. The 2,3-^13^C_2_ lactate ‘signature’ of characteristic doublets (Figure 1) in the ^13^C NMR spectrum is evidence that the ^13^C-^13^C bond stays intact, because the probability of two ^13^C atoms occurring next to each other naturally without exogenous double-labelling is 0.01%. Thus, the 8 mmol/L 1,2-^13^C_2_ glucose delivered directly to the brain’s extracellular space by microdialysis catheters was taken up by brain cells, metabolised by glycolysis as the main pathway into lactate, and this ^13^C-labelled lactate was exported into the brain extracellular space, where it was recovered by microdialysis catheters for analysis by ISCUS*flex* (Figure 2) and ^13^C NMR (Figure 3). This concurs with our previous microdialysis studies using 1,2-^13^C_2_ glucose at 2 mmol/L and 4 mmol/L concentrations.^3,11^ Also as previously, a lower enrichment of singly-labelled lactate (3-^13^C lactate) showed the PPP to be a lesser contributor to lactate production than glycolysis (2,3-^13^C_2_ lactate) from the exogenously-supplied 1,2-^13^C_2_ glucose.^11^

### Glucose supplementation at 4 mmol/L: brain biochemistry compared to baseline

Eleven patients, who received microdialysis perfusion supplemented with 4 mmol/L 1,2-^13^C_2_ glucose, possessed complete baseline (*both* pre- and post-supplementation) ISCUS*flex* datasets (Supplementary Table 1). Mean baseline concentrations were glucose 1.22 mmol/L, lactate 3.26 mmol/L and pyruvate 113 µmol/L. Glucose supplementation increased microdialysate glucose by 234%, produced a mean 19% increase (versus baseline) in extracellular lactate (*p* *<* *0.0001*, *lmer*) and 17% increase in extracellular pyruvate (*p < 0.0001*, *lmer*). A mean 5% increase in LPR (*p = 0.002*, *lmer*), was unlikely to be biologically or clinically significant (Figure 2). Glycerol rose by a mean 8% (*p < 0.0001*, *lmer*), but there was no significant change in extracellular glutamate.

### Glucose supplementation at 8 mmol/L: brain biochemistry compared to baseline

Twelve patients received microdialysis with CNS perfusion fluid supplemented with 8 mmol/L 1,2-^13^C_2_ glucose. One of these 12 patients also had a second microdialysis catheter simultaneously supplemented with 4 mmol/L 1,2-^13^C_2_ glucose. Eight of these 12 patients had complete ISCUS*flex* datasets comprising pre-supplementation, during-supplementation, and post-supplementation. Mean baseline concentrations were glucose 1.61 mmol/L, lactate 2.56 mmol/L, and pyruvate 98 µmol/L. Compared to baseline perfusion with plain (unsupplemented) CNS perfusion fluid, glucose supplementation increased microdialysate glucose by 370%, but did not result in a statistically significant change (*lmer*) in extracellular lactate concentration (average 2% decrease, *p = 0.17*) or pyruvate (average 2% decrease, *p = 0.12*). Similarly, there was no significant change in LPR (average 1.5% increase, *p = 0.17*, *lmer*) (Figure 2). There was a statistically significant 15% increase in glycerol (*p < 0.0001*, *lmer*) and 21% fall in glutamate (*p < 0.0001*, *lmer*).

### Glucose supplementation in the neuroglycopenic brain

Seven patients had a low baseline extracellular brain glucose, defined as <0.8 mmol/L.^8,23^ Analysis of microdialysis (ISCUS*flex*) data from neuroglycopenic patients – 3 patients supplemented with 8 mmol/L 1,2-^13^C_2_ glucose and 4 patients supplemented with 4 mmol/L 1,2-^13^C_2_ glucose – revealed a similar pattern as supplementation in the 4 mmol/L 1,2-^13^C_2_ glucose group as a whole: a mean 15% increase in lactate (*p < 0.001*), 15% increase in pyruvate (*p < 0.001*), and only 4% increase in LPR (*p = 0.003*) (Figure 2).

### NMR analysis of microdialysates from 8 mmol/L 1,2-^13^C_2_ glucose perfusion

Lactate is an important glucose metabolite found at mmol/L concentrations in cerebral extracellular fluid in health and TBI.^11^ The mean percentage of lactate labelled with ^13^C at the C3 position in recovered microdialysates was 17.6% (s.d. 5.4%). This comprised 14.7% (s.d. 4.9%) doubly-labelled (2,3-^13^C_2_ lactate, 0.39 mmol/L) derived from glycolysis, and 2.0% (s.d. 0.7%) singly-labelled (3-^13^C lactate, 0.03 mmol/L, 1.1% ^13^C natural abundance subtracted) derived from PPP metabolism (Figure 3, Table 2 and Supplementary Figure 1). For further explanation of PPP biochemistry, see Carpenter et al.^7^ and Jalloh et al.^11^

**Table 2.** NMR results.

	Glycolytic 2,3- ^13^C_2_ lactate (calculated from C3 doublet)		PPP 3- ^13^C lactate^a^ (calculated from C3 singlet)		Total ^13^C-lactate^a^ C3 enrichment (calculated from C3 singlet + C3 doublet)		Percentage of total ^13^C at C3 of lactate that is derived from PPP	Ratio of PPP 3-^13^C lactate to glycolytic 2,3-^13^C_2_ lactate
ID	Conc. mmol/L	FE %	Conc. mmol/L	FE %	Conc. mmol/L	FE %	%	Ratio
GL-01	0.15	8	0.015	0.8	0.18	8.9	9.1	1:10.0
GL-02	0.27	19	0.040	2.9	0.32	21.9	13	1:6.8
GL-05	0.61	13	0.081	1.7	0.73	14.7	12	1:7.5
GL-07	0.83	13	0.130	2.1	1.00	15.1	14	1:6.4
GL-08	0.24	14	0.020	1.2	0.27	15.2	8	1:12.0
GL-09	0.19	10	0.022	1.2	0.23	11.2	10	1:8.6
GL-11	0.22	13	0.031	1.8	0.26	14.8	12	1:7.1
GL-12	0.22	12	0.039	2.2	0.28	14.2	15	1:5.6
GL-13	0.38	18	0.053	2.5	0.46	20.5	12	1:7.2
GL-14	0.6	15	0.084	2.1	0.72	16.9	12	1:7.1
GL-15	0.63	27	0.073	3.1	0.72	30.1	10	1:8.6
GL-16	0.39	15	0.049	1.9	0.46	16.9	11	1:8.0
Mean	0.39	15	0.053	2.0	0.47	16.7	12	1:7.9

Results of high-resolution NMR analysis of microdialysate (each patient’s NMR sample was a pool of 24 × 1 h vials) during supplementation with 8 mmol/L 1,2-^13^C_2_ glucose. Total number (n) of patients whose data were included in this table was 12. FE: fractional enrichment – see Methods for details of calculation.

^a^The 3-^13^C lactate fractional enrichment results are presented after background subtraction to remove the contribution from natural abundance background ^13^C that is 1.1% of all carbon atoms. 2,3-^13^C_2_ lactate results are not background-subtracted as the probability of two natural ^13^C atoms occurring next to each other by chance is only 0.01% (=1.1% × 1.1%).

NMR spectroscopy showed no evidence for TCA cycle metabolism of 1,2-^13^C_2_ glucose supplemented at 8 mmol/L. Notably, doubly-labelled ^13^C glutamine was undetectable in microdialysates from all 12 patients with NMR analysis; only two of these showed singly-labelled glutamine but not above background ^13^C natural abundance.

## Discussion

Here we have shown that the traumatised human brain can metabolise additional glucose to a limited degree when delivered directly into the brain extracellular fluid via a microdialysis catheter. Glucose supplementation may be a useful strategy in TBI patients in certain circumstances, discussed below.

### Significance of ^13^C-labelling in metabolites

The substrate 8 mmol/L 1,2-^13^C_2_ glucose was metabolised to 2,3-^13^C_2_ lactate, detected by NMR in the recovered microdialysates, providing clear unambiguous evidence of glycolysis, with a smaller amount of 3-^13^C lactate indicating metabolism via the PPP as a minor route for lactate production.

Despite this unambiguous evidence of glycolytic (and PPP) metabolism of 1,2-^13^C_2_ glucose, there was no detectable ^13^C NMR evidence for TCA cycle metabolism – notably no doubly-labelled glutamine was identified, while singly-labelled glutamine was only at natural-abundance background level, similar to previous studies employing lower concentrations of 1,2-^13^C_2_ glucose.^3,11^

The lack of change in ISCUS*flex*-measured extracellular metabolites (whereby labelled and unlabelled molecules are not differentiated) during perfusion with 8 mmol/L 1,2-^13^C_2_ glucose suggests several explanations, below.

### Limited effect on glycolytic activity – changes in extracellular lactate and pyruvate measured by bedside ISCUSflex

Predictably, 1,2-^13^C_2_ glucose supplementation caused a marked, significant rise in microdialysate levels of glucose measured on the ISCUS*flex* bedside analyser. Supplementation with 4 mmol/L glucose produced significant rises in lactate (19%) and pyruvate (17%), suggesting increased glycolytic activity and possibly PPP too. However, supplementation with 8 mmol/L glucose did not increase lactate and pyruvate concentrations further.

Probably these different responses are at least partly due to patient differences. Baseline glucose was 32% lower in the 4 mmol/L supplementation group (1.22 mmol/L) than the 8 mmol/L supplementation group (1.61 mmol/L); while lactate and pyruvate were 14% and 21% higher, respectively. Supplementation of additional glucose appeared less effective at increasing glycolysis in patients with an ‘already-high’ baseline glucose, in contrast to patients with lower baseline glucose. This is evident in subgroup analysis of neuroglycopaenic patients, where there were modest, statistically significant increases in lactate, pyruvate and LPR when supplemented with 1,2-^13^C_2_ glucose via microdialysis, regardless of supplementation concentration. This ‘neuroglycopaenic’ subgroup nevertheless showed a similar pattern of metabolite labelling to the study cohort overall, wherein ^13^C labelling in lactate indicated glycolysis and PPP, but no detectable ^13^C labelling in glutamine.

Additionally, the relatively high baseline lactate and pyruvate in patients who received 4 mmol/L glucose supplementation may indicate tissue able to metabolise glucose relatively freely – and thus can metabolise additional glucose when supplemented. Contrastingly, the relatively lower baseline lactate and pyruvate in those patients who subsequently received 8 mmol/L glucose may represent underlying relative impairment of cerebral glycolysis (and PPP), hindering upregulation of metabolism when given excess glucose.

The biochemical behaviour pattern of TBI patients who received 8 mmol/L glucose concurs with earlier findings by Jalloh *et al.* in their non-TBI “normal brain” group, where 4 mmol/L glucose elicited a proportionate rise in ISCUS*flex*-measured glucose but no significant changes in lactate or pyruvate, and a minor (statistically significant) increase in LPR.^11^ Their statistical analysis utilised pooled data rather than individual biochemistry.^11^ Although statistically non-significant (possibly because of low number of patients), median levels of for lactate and pyruvate increased with glucose supplementation in non-TBI “normal brain”.^11^ Conversely, in TBI patients, lactate and pyruvate did not increase with glucose supplementation.^11^ The ability of TBI brain to metabolise additional glucose via glycolysis and the PPP might depend on its baseline metabolic ‘health’ that may be poorer in TBI patients.

The self-limiting nature of glycolysis may also be relevant. Cells take up glucose via specific transporters^24,25^ then phosphorylate it with hexokinase, locking it inside the cell in the first step of glycolysis. Normally the rest of glycolysis would follow, leading to pyruvate (and then lactate and/or to acetyl-CoA and TCA cycle). However, if there is too much glucose the hexokinase is product-inhibited by glucose-6-phosphate and the cell stops taking up more glucose, thereby limiting glycolysis.^26^ This may be relevant to the present study, particularly with high (8 mmol/L) glucose supplementation. The potentially deleterious effect of severe hyperglycaemia in the brain after TBI was reported^24,25^ in studies considering elevated serum glucose rather than increased brain interstitial glucose. Such adverse effects might manifest at least partly through effects on brain vasculature, less relevant to this study. Nevertheless, a comprehensive study of graded hyperglycaemia in humans and rabbits found that more extreme hyperglycaemia may cause neuronal injury and suppress astrocyte activation in the frontal cortex, whereas moderate hyperglycaemia may not.^27^ Furthermore, a positive statistical relationship between microdialysate glucose concentration and mortality was reported in TBI patients, alongside a negative relationship between pyruvate concentration and mortality. This concurs with the idea that abnormally high levels of brain extracellular glucose may exist because injured brain cells are poorly able to metabolise it.

A caveat to our present study’s technique is that glucose given directly into the brain extracellular fluid might differ in its subsequent processing compared to glucose delivered via the circulation (e.g. intravenous glucose, discussed below).

### Extracellular ^13^C lactate – dilution, glycolysis, and the pentose phosphate pathway

Supplementing the brain extracellular space with 8 mmol/L 1,2-^13^C_2_ glucose yielded no evidence of TCA cycle metabolism (the spin-out product ^13^C-labelled glutamine was undetected) and there were no significant changes in total labelled plus unlabelled concentrations of lactate and pyruvate. However, the ^13^C-labelled glucose was clearly metabolised by glycolysis and the PPP, evidenced by recovered ^13^C-lactate with the diagnostic labelling patterns in microdialysates. This implies that the metabolism of the exogenous ^13^C labelled glucose was seemingly *in place* of that of endogenous unlabelled glucose, discussed below. The ^13^C NMR results appear to reveal the ceiling of cerebral glycolytic metabolism of this cohort.

When glycolysis breaks down one mole of 1,2-^13^C_2_ glucose, it produces 1 mole of 2,3-^13^C_2_ lactate and 1 mole of unlabelled lactate (via their respective precursor pyruvates), thereby self-diluting the labelled lactate. Thus, the theoretical maximum level of enrichment of lactate at the region of interest (ROI) addressed by the microdialysis catheter assuming exposure to 1,2-^13^C_2_ glucose at 99% enrichment is 49%. However, when baseline extracellular unlabelled lactate is considered, this may be expected to fall to around 44% (assuming an unlabelled baseline glucose concentration of 1 mmol/L). The mean enrichment level observed in the C3 position of lactate (combined glycolytic 2,3-^13^C_2_ lactate and PPP 3-^13^C lactate) in this study was under half this, at 16.7%. The highest individual level of ^13^C enrichment observed in lactate was 30%, which is over three-fifths of the theoretical maximum level of enrichment of 49% if the ROI addressed by the microdialysis catheter was exposed solely to 1,2-^13^C_2_ glucose at 99% enrichment. This relative paucity of lactate ^13^C enrichment was unexpected given the high concentration of glucose perfused, with potential explanations below.

**Figure 4.**
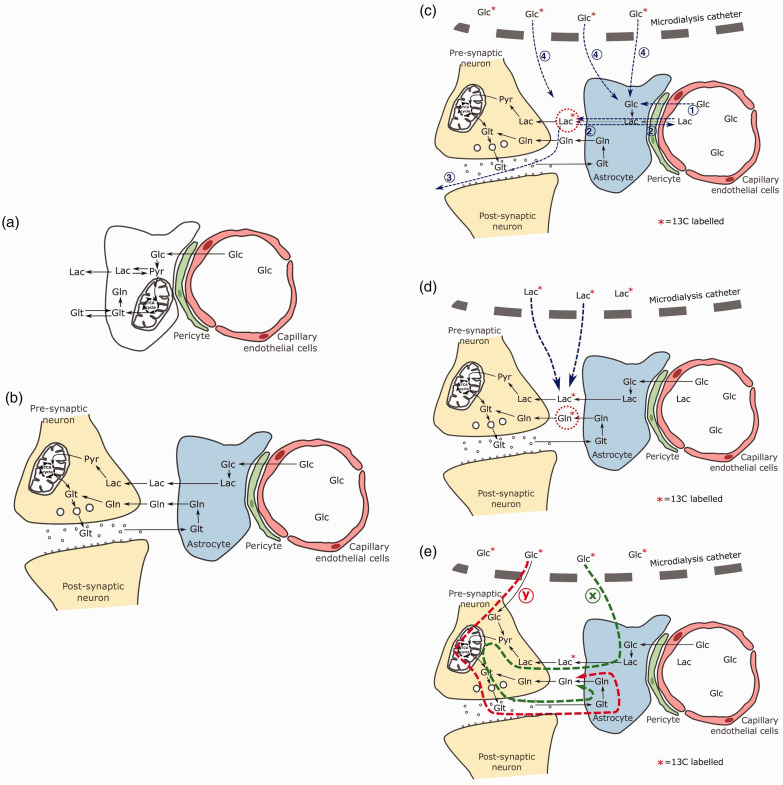
Proposed schematics of intercellular metabolic pathways including astrocyte-neuron lactate shuttle hypothesis and explanations for low ^13^C lactate enrichment and absent ^13^C glutamine enrichment – indicating the importance of extracellular lactate in glutamine production. *Panel a:* Simplified single-cell metabolism of glucose. Glucose from the vasculature is metabolised to form pyruvate by glycolysis or the pentose phosphate pathway. Pyruvate may then either be converted to lactate and exported extracellularly, or incorporated into the mitochondrial TCA cycle, leading to ATP synthesis (oxidative phosphorylation). Glutamate is also produced as a spin-off from alpha-ketoglutarate for release into the synaptic cleft as a neurotransmitter, before being taken up and converted to glutamine. *Panel b:* Simplified schematic of metabolic trafficking in the brain according to the astrocyte-neuron astrocyte shuttle hypothesis.^34^ Glucose from the vasculature is metabolised by astrocytes to lactate, then exported to local neurons via the extracellular fluid. Lactate is incorporated into the neuronal mitochondrial TCA cycle, leading to ATP synthesis and glutamate production for release into the synaptic cleft as a neurotransmitter. Leftover released glutamate is taken up by adjacent astrocytes, converted to glutamine and delivered back to neurons via the extracellular fluid. Extracellular lactate and glutamine are important extracellular mediators in this model. *Panel c:* Potential explanations for relatively low ^13^C lactate enrichment (16.7%; 0.45 mmol/L) despite high concentration ^13^C glucose supplementation via microdialysis: (1) Glucose may more readily be absorbed by glia and neurons from capillaries via its natural route, rather than through the extracellular space. (2) Exchange of ^13^C lactate with unlabelled lactate from the systemic circulation. (3) Local diffusion of ^13^C lactate away from the region surrounding the catheter. (4) Insufficient rate of delivery of ^13^C glucose by catheter compared to the cellular metabolic rate of glucose. *Panel d:* Microdialysis delivered ^13^C labelled lactate yields ^13^C labelled glutamine. Delivery of modest concentrations ^13^C lactate (4 and 8 mmol/L) by microdialysis catheter results in ^13^C labelling of glutamine.^3,16^ *Panel e:* Extracellular lactate is an important step in the brain’s production of glutamine. Delivery of ^13^C glucose by microdialysis catheter does not result in ^13^C labelling of glutamine, but does produce low percent enrichment ^13^C lactate labelling (9-30%). This suggests that additional dilution of the ^13^C label occurs at the point of extracellular lactate, so pathway ‘x’ that shuttles lactate between cells (coloured green) is more likely to represent the dominant pathway of glutamine production in the traumatised human brain than pathway ‘y’, coloured red, that does not involve extracellular lactate. Pathway ‘y’ would have expected to produce ^13^C labelled glutamine when ^13^C glucose is perfused at high concentrations, but not when ^13^C lactate is perfused. *Glc, glucose; Lac, lactate; Pyr, pyruvate; Glt, glutamate; Gln, glutamine.*

Microdialysis delivery of 8 mmol/L 1,2-^13^C_2_ glucose was used to create focal supra-physiological cerebral concentrations of glucose in the brain extracellular fluid. However, this might not entirely reproduce the process of glucose delivery from the circulation. The brain has an extensive vascular supply, and astrocyte end-feet are firmly attached to the endothelial cells of the blood capillaries constituting the tight junctions of the blood-brain barrier (Figure 4). Glucose uptake from blood into brain is controlled via specific transporters.^24,25^ Glucose transfer from blood in the capillary into the astrocytes directly adjoining the endothelial cells might conceivably be more effective at feeding glucose into the astrocytes than direct uptake from the extracellular fluid (Figure 4), perhaps explaining the discrepancy between observed and theoretical ^13^C lactate enrichment.

Other likely sources of dilution include active or passive exchange with endogenous lactate from the systemic circulation, and passive diffusion with adjacent brain. Indeed, arteriovenous difference measurements have shown that the brain undergoes periods of net uptake and net export of lactate.^7^

Glycolysis is the primary metabolic path for glucose metabolism in the brain, generating 2 moles of ATP per mole of glucose, whereas the PPP is an alternative pathway (a complex detour around some of the steps of glycolysis) that neither consumes nor produces ATP, but sacrifices some of the brain’s glucose for the sake of producing intermediates for protecting and repairing the brain^7,11^ (Supplementary Figure 1). This was confirmed in the 7.9:1 mean ratio of 2,3-^13^C_2_ lactate to 3-^13^C lactate in this study (Table 2). There was a close positive linear correlation between 2,3-^13^C_2_ lactate and 3-^13^C lactate (Pearson’s r^2^ = 0.73 for fractional enrichments and r^2^ = 0.94 for concentrations) (Figure 3(b) and (c) respectively). Thus, the minor PPP lactate production mirrored the major glycolytic lactate production. The PPP may be increased after TBI, evidenced by rodent studies of moderate-severe TBI,^28,29^ but a microdialysis study in human non-TBI controls and TBI patients suggested a more varied response in the ratio of PPP to glycolytic metabolism, with the tendency for more patients to have a reduction in their relative PPP metabolism.^11^

### TCA cycle activity – absence of recovered ^13^C glutamine

No evidence of supplemented 1,2-^13^C_2_ glucose metabolism via the TCA cycle was found using ^13^C NMR analysis of microdialysis samples as ^13^C glutamine was only found at its natural-abundance background level. Previous microdialysis studies in TBI and non-TBI surgical patients in which ^13^C glucose was delivered at lower concentrations to the human brain similarly have failed to produce ^13^C glutamine enrichment.^3,11^ Conversely, clear evidence of TCA cycle ^13^C labelling in glutamine *was* found when the three other substrates 2,3-^13^C_2_ succinate, 2-^13^C acetate, and 3-^13^C lactate were perfused by microdialysis (Figure 4).^3,16,17^

Literature from magnetic resonance studies shows that intravenous supplementation of ^13^C-labelled glucose can be metabolised by the brain *in-vivo* via the TCA cycle, evidenced by detection of ^13^C-label in glutamine and glutamate. Those studies address whole brain tissue, dominated by the *intracellular* compartment, thereby differing from microdialysis that samples *extracellular* molecules. Studies in healthy human volunteers and animals receiving intravenous ^13^C-glucose have shown ample evidence of TCA cycle activity by cerebral ^13^C MRS *in-vivo*,^30,31^ however this has not yet been performed in TBI patients. In animals, intravenous ^13^C-glucose both in TBI-models and controls showed TCA cycle evidence, by *ex-vivo* NMR analysis of ^13^C in brain tissue extracts, showing ^13^C labelling in glutamate and/or glutamine.^28,29^

Contrary to evidence from brain tissue, evidence of TCA cycle production of ^13^C glutamine from microdialysis delivery of ^13^C glucose seems consistently lacking, previously ascribed to ^13^C label dilution by endogenous material given the lower glucose concentrations perfused, as many biosynthetic steps and intermediates are involved in the pathway between glucose and glutamate-glutamine.^3,11^ TCA cycle ^13^C labelling of glutamine by microdialysis delivery of other substates – 2,3-^13^C_2_ succinate,^17^ 2-^13^C acetate,^3^ and 3-^13^C lactate^3,16^ – is explicable because these three substrates are closer (in the number of main biosynthetic steps) to the TCA cycle than glucose – succinate is itself a TCA cycle intermediate, acetate is one step from the TCA cycle, and lactate is three steps from the TCA cycle. Microdialysis delivery of 3-^13^C lactate (8 mmol/L) with 99% fractional enrichment resulted in ^13^C labelling in the product glutamine, showing NMR singlets for C4, C3 and C2 with maximal fractional enrichments of 15.8%, 11.9% and 7.5% respectively.^16^ Contrastingly, in the present study, microdialysis administration of 1,2-^13^C_2_ glucose (8 mmol/L,) with a 99% fractional enrichment only resulted in 2,3-^13^C_2_ lactate and 3-^13^C lactate with maximal fractional enrichments of 31% and 3.6% respectively (average total ^13^C lactate enrichment 16.7%; 0.45 mmol/L), and no detectable ^13^C-labelling in glutamine. Thus, despite 1,2-^13^C_2_ glucose administration at a supra-physiologically high concentration and high ^13^C fractional enrichment, insufficient enrichment was attained at the metabolic stage of lactate, and consequently undetectable ^13^C-labelling in glutamine.

Another possible explanation of lack of ^13^C labelling in glutamine is mitochondrial dysfunction. In an earlier study of 3-^13^C lactate (8 mmol/L) given by microdialysis, most of the patients showed TCA cycle metabolism evident from ^13^C labelling in glutamine, but a few were ‘non-responders’ who gave no ^13^C-glutamine production, attributed to mitochondrial dysfunction.^16^ However, it is unlikely that *all* of the patients tested with 1,2-^13^C_2_ glucose in the present and previous study^11^ would have been non-responders because of mitochondrial dysfunction, particularly as many of them had baseline LPR < 25.

Patients with PbtO_2_ monitoring showed mean 27 mmHg, with none <15 mmHg, thus not hypoxic, and all 20 patients in the present study were treated with the same protocol-driven therapy aimed at adequate cerebral perfusion. Thus, the absence of TCA cycle ^13^C labelling in glutamine is unlikely to be due to oxygen deficiency. Hyperoxia may suppress cellular glycolysis;^32^ its effects are variable on cerebral microdialysis lactate and LPR, and may increase cerebral oxygen extraction in acute severe TBI patients.^33^ However, none of the patients in our study demonstrated cerebral hyperoxia (highest PbtO_2_ 38 mmHg; patient GL-13), nor were they exposed to extreme arterial hyperoxaemia (PaO_2_ ≤17 kPa; except patient GL-05 whose mean PaO_2_ was 19 kPa).

### Extracellular lactate as a key step in brain metabolism

The lack of ^13^C glutamine detected in this study suggests that extracellular lactate is an important intermediate step in brain glucose metabolism (Figure 4). Since we achieved an average 16.7% enrichment of ^13^C lactate by glycolysis of ^13^C glucose, similar enrichment would be expected in equivalent downstream metabolites within a single cell ‘independently’ performing all steps of glycolysis and the TCA cycle. However, if the ^13^C label was further diluted by its export as ^13^C lactate to the extracellular environment where it mixed with unlabelled lactate, before a population of cells metabolised it to glutamine, the final ^13^C glutamine enrichment may be insufficient to detect. When 3-^13^C lactate (99% enrichment) is supplemented directly at 4 or 8 mmol/L concentration, ^13^C glutamine is detected,^3,16^ whereas when 3-^13^C lactate is delivered *indirectly* (at average 0.45 mmol/L concentration, 16.7% enrichment) in this study by supplementation with 1,2-^13^C_2_ glucose at 8 mmol/L ^13^C, glutamine is not detected.

Debate exists whether astrocytic glycolysis-derived lactate is the preferred energy substrate for neurons as in the astrocyte-neuron lactate shuttle hypothesis (ANLSH – see below), or whether both astrocytes and neurons independently metabolise glucose per their needs.^34–36^ Supporting the ‘independent model’ (vs. ANLSH) is a kinetic metabolic modelling study in rats.^35^

In the ANLSH (Figure 4),^2,34,36^ metabolic trafficking between astrocytes and neurons can be summarised as follows.^3^ Glucose from the vasculature is taken up by astrocytes that metabolise it by glycolysis to form lactate that is exported out of the astrocyte, then taken up by neurons that metabolise it via the TCA cycle. A portion of the TCA cycle intermediate alpha-ketoglutarate is converted into the spin-off product glutamate released from the pre-synaptic neuron into the synaptic cleft where it can stimulate glutamate receptors on the post-synaptic neuron. Extracellular glutamate is taken up by astrocytes, converted into glutamine^37^ and exported into the extracellular fluid, then taken up by neurons and converted back into glutamate.^38^ Outside of the synaptic cleft, extracellular glutamate concentrations are typically low (1–20 µmol/L, rising to 100–200 µmol/L in ischaemia); glutamine concentrations are typically 400–1000 µmol/L.^39,40^

Both glucose metabolic models – ‘independent’ and ANLSH – feature glutamate-glutamine cycling between neurons and astrocytes.^35^ Our study’s lack of ^13^C glutamine when microdialysis-delivered 1,2-^13^C_2_ glucose is the substrate is compatible with the ANLSH – implying that lactate is the favoured substrate for the neuronal TCA cycle.^41^ Therefore, when 1,2-^13^C_2_ glucose is the substrate (even when supra-physiological and highly enriched) the ensuing 2,3-^13^C_2_ lactate becomes too diluted with unlabelled lactate. Moreover, the astrocytic glycolysis-derived lactate is not only self-diluted with the lactate from the unlabelled half of the 1,2-^13^C_2_ glucose molecule (C4, C5 and C6), but also exits into the extracellular pool, where it can mix with endogenous lactate, and is then taken up by neurons for TCA cycle processing resulting in glutamine. Furthermore, astrocytes/glia can themselves synthesise glutamate de novo via the TCA cycle alpha-ketoglutarate, and ensuing glutamate converted to glutamine by the same astrocytes/glia.^42,43^

In future, self-dilution could be overcome by using uniformly ^13^C labelled glucose. Glycolysis then yields two molecules of 1,2,3-^13^C_3_ pyruvate, then forms 1,2,3-^13^C_3_ lactate, with no self-dilution. This would facilitate detecting TCA cycle products, albeit with more complex ^13^C NMR spectral splitting patterns thus smaller peaks.

### Clinical relevance of glucose delivery and alternative fuels in TBI

The ISCUS*flex* and ^13^C results of this and previous^11^ studies together suggest that glucose supplementation may have limited utility in human TBI. Evidence for glycolysis (and minor PPP) rather than TCA cycle were found, but no shift towards high-ATP-yield oxidative TCA cycle metabolism (no decrease in LPR and no detectable ^13^C glutamine). Furthermore, glycolysis, which produces 2 moles of ATP per mole of glucose metabolised, only increased in patients with either low baseline glucose, or evidence of functioning metabolism producing higher concentrations of extracellular lactate and pyruvate at baseline.

A caveat is that glucose was delivered directly into the brain extracellular fluid, which may be less efficient than by its ‘natural’ route from the systemic circulation. Blood glucose concentration and glycaemic control normally influence brain glucose, though this relationship may be weaker in injured brain.^12,14,44–49^ In the present study there was a non-significant positive trend (Pearson r^2^ = 0.17, p = 0.08) between blood glucose and brain microdialysate (ISCUS*flex*) glucose concentrations during the microdialysis supplementation period, but no significant relationships between blood glucose concentrations and brain microdialysate (ISCUS*flex*) concentrations of lactate, pyruvate or LPR. Elsewhere, blood glucose levels reportedly influenced brain metabolism in TBI patients, evidenced by arterial-jugular venous difference measurements^50^ and cerebral microdialysis.^51,52^ In these, blood glucose levels 6–9 mmol and cerebral microdialysate glucose 1–5 mmol were associated with minimizing the corresponding microdialysate LPR and glutamate concentration. High microdialysate glucose >5 mmol was associated with high glutamate, while low microdialysate glucose <1 mmol was associated with high LPR, in a small study.^51^ Moreover, data from 619 TBI patients also showed high microdialysate LPR associated with low microdialysate glucose.^53^ In the present small study, there was no significant relationship between microdialysate LPR and microdialysate glucose.

Delivering additional glucose to the whole brain using intravenous infusions to achieve iatrogenic hyperglycaemia may have risk. A large study of general critical care patients (mainly non-TBI) revealed worse outcome in those with loosely-controlled serum glucose,^54^ but unconfirmed in a later, similar-sized study.^55^ A more recent meta-analysis in TBI patients found that tight glucose control was associated with better neurological outcome than loose.^56^ Conversely, other TBI studies suggested loose glucose control was better than tight.^12,14^ An observational study of 86 TBI patients^57^ with blood glucose targeted between 6-8 mmol/L (using an insulin sliding scale) achieving a mean 6.6 (SD 1.1) mmol/L concluded that increased blood glucose may impair cerebrovascular reactivity, suggesting mechanistic linkage between increased blood glucose and poorer outcome post-TBI. Defining the optimal ranges of both cerebral and plasma glucose is beyond the scope of the present study and requires well-designed prospective studies. However, the present study does indicate that adding glucose focally to the extracellular space does not cause a major change in LPR: administration of 4 mM or 8 mM glucose via the microdialysis catheter resulted in mean LPR increases of 5% and 1.5% respectively.

Discussion grows on whether substrates other than glucose have therapeutic potential in TBI. Debate exists^58^ on the suitability of administering exogenous lactate for TBI therapy,^59–61^ although it is clearly metabolised via the TCA cycle,^16^ and its metabolism appears unsuppressed after TBI.^4^ However, the administration time post-injury may be important.^5^ Evidence increases that succinate may support metabolism of the acutely traumatised human brain.^17,62,63^ Whether such alternative substrates have therapeutic roles need more extensive comparative studies against glucose.

## Conclusions

Using a reductionist approach, directly delivering glucose into the extracellular space of the traumatised human brain, we have shown limited ability of the brain to metabolise *additional* glucose in only some patients. This appears to be principally determined by a patient’s underlying disturbance in their injured brain’s ability to metabolise glucose, or a saturation of their existing metabolic pathways, as its utilisation may be greater in cases of relative neuroglycopaenia.

As expected, we found that supplemented ^13^C glucose yielded extracellular ^13^C lactate derived from both glycolysis and the PPP. However, ^13^C lactate did not attain sufficient enrichment to allow subsequent detection of TCA cycle spin-out products, thus no ^13^C glutamine was detected – despite it being found in studies that directly supplemented ^13^C lactate or ^13^C acetate.^3,16^ Our findings suggest dilution of the ^13^C label with extracellular endogenous metabolites occurs after glycolysis, and during glycolysis where self-dilution with unlabelled carbons from the breakup of 1,2-^13^C_2_ glucose forming a 1:1 mixture of labelled and unlabelled pyruvate occurs. The metabolism of glucose and its products appears likely to be shared between multiple cells *in-vivo*, rather than occurring to completion in a single ‘stand-alone’ cell, and extracellular lactate is an important step for such appearance of extracellular glutamine in the traumatised brain. Further studies in TBI patients with intravenous ^13^C-labelled glucose and monitoring by *in-vivo* MRS and cerebral microdialysis are warranted, to address respectively the intracellular and extracellular compartments.

## Supplemental Material

sj-pdf-1-jcb-10.1177_0271678X231173584 - Supplemental material for High-physiological and supra-physiological 1,2-^13^C_2_ glucose focal supplementation to the traumatised human brain Click here for additional data file. Supplemental material, sj-pdf-1-jcb-10.1177_0271678X231173584 for High-physiological and supra-physiological 1,2-^13^C_2_ glucose focal supplementation to the traumatised human brain by Matthew G Stovell, Duncan J Howe, Eric P Thelin, Ibrahim Jalloh, Adel Helmy, Mathew R Guilfoyle, Peter Grice, Andrew Mason, Susan Giorgi-Coll, Clare N Gallagher, Michael P Murphy, David K Menon, T Adrian Carpenter, Peter J Hutchinson and Keri LH Carpenter in Journal of Cerebral Blood Flow & Metabolism
