# Supplementary material for: High-physiological and supra-physiological 1,2-13C2 glucose focal supplementation to the traumatised human brain
Source: J Cereb Blood Flow Metab. 2023 May 8;43(10):1685–701. doi: 10.1177/0271678X231173584 (PMC10581237; doi:10.1177/0271678X231173584)
Supplement: sj-pdf-1-jcb-10.1177_0271678X231173584 - Supplemental material for High-physiological and supra-physiological 1,2-13C2 glucose focal supplementation to the traumatised human brain [file sj-pdf-1-jcb-10.1177_0271678X231173584.pdf]

Matthew G. Stovell et al. Supplemental Information

Supplemental Figure 1.

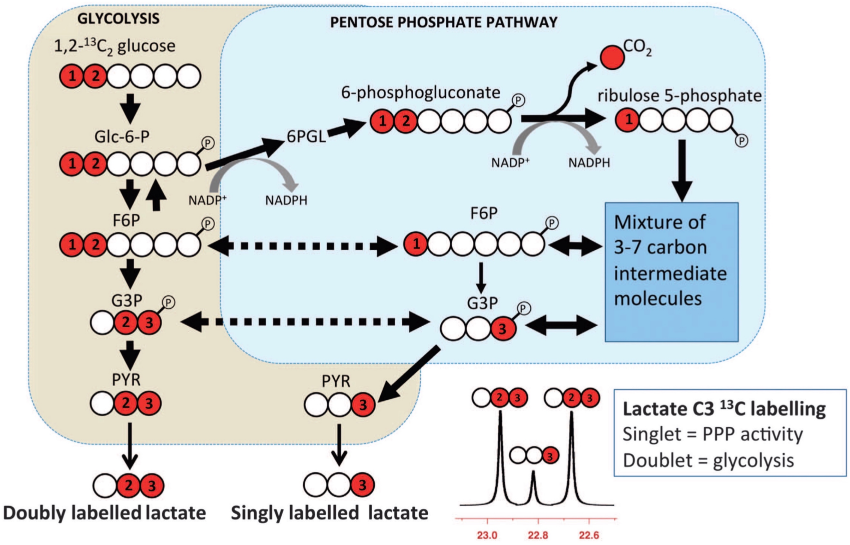

Supplementary Figure 1. Schematic of ^{13}C labelling in glycolysis and the pentose phosphate pathway. Simplified schematic of steps in glycolysis and the pentose phosphate pathway (PPP) showing ^{13}C labelling patterns resulting from 1,2-$^{13}C_2$ glucose supplementation. ^{13}C atoms indicated by red fills. Glc-6-P, glucose-6-phosphate; 6PGL, 6-phosphogluconolactone; F6P, fructose-6-phosphate; G3P, glyceraldehyde-3-phosphate; PYR, pyruvate. Figure originally published by KLH Carpenter et al. in European Journal of Pharmaceutical Sciences 57 (2014) 87–97, under a Creative Commons Attribution License (CC BY).

Supplemental Table 1. Patient demographics

ID	Age	Sex	Mechanism Injury	GCS	Brain Injury	ICP mmHg	CPP mmHg	$P_{bt}O_2$ mmHg	4mmol ISCUS Data	8mmol ISCUS Data	8mmol NMR Data	Days from TBI	Cath Latr
**GL-01**	65	M	Assault	E1V1M2	ASDH	14	68	NA	X	X	X	1	Ipsi
**GL-02**	39	M	Assault	E1V2M3	ICH/EDH/contusions	NA	NA	NA			X	3	Ipsi
**Gl-03**	33	F	RTC (ped-car)	E1V2M2	Contusions	13	77	NA	X			11	Ipsi
**GL-04**	37	M	Fall	E1V1M4	ASDH/contusions	11	75	43	X			2	Cont

**GL-05**	23	M	RTC (car-wall)	E1V1M5	Contusions	19	84	20	X		X	9	Ipsi
**GL-06**	19	F	Fall (horse)	E1V1M3	Contusions	9	75	38	X			2	Cont
**GL-07**	39	M	Fall	E1V1M5	Contusions	NA	NA	NA	X		X	4	Cont
**GL-08**	44	M	RTC (cycl-car)	E1V1M2	ASDH/contusions	22	79	NA		X	X	1	Ipsi
**GL-09**	55	F	RTC (cycl-car)	E1V1M5	ASDH	6	80	30		X	X	2	Cont
**GL-10**	41	F	RTC (ped-car)	E2V2M5	EDH/ASDH/contusions	7	81	24	X			5	Cont
**GL-11**	32	M	RTC (car-wall)	E1V2M5	ASDH/contusions	5	80	NA		X	X	1	Cont
**GL-12**	16	F	RTC (car-wall)	E1V1M2	DAI	9	71	NA		X	X	1	Ipsi
**GL-13**	32	M	Assault	E2V1M4	ASDH	13	74	NA		X	X	2	Ipsi
**GL-14**	32	M	RTC	E1V2M4	ASDH	19	73	16		X	X	2	Ipsi
**GL-15**	19	M	Fall	E1V1M2	ASDH	9	76	NA		X	X	2	Cont
**GL-16**	40	M	Fall	E4V1M5	ASDH/EDH/contusions	NA	NA	NA			X	2	Cont
**GL-17**	27	M	RTC	7	Contusions	NA	NA	NA	X			1	Cont
**GL-18**	53	F	RTC	6	DAI	NA	NA	NA	X			1	Ipsi
**GL-19**	37	M	RTC	8	Contusions	NA	NA	NA	X			2	Ipsi
**GL-20**	28	M	RTC	3	ASDH/contusions	NA	NA	NA	X			4	Ipsi

Table indicates whether full ISCUS*flex* and/or high-resolution NMR data were available, and hence used for analysis (‘x’ indicates available data). Total number (n) of patients included in this table was 20. ICP, CPP and $PbtO_2$ data represents supplementation period median values. Days from TBI denotes time interval between injury and period of supplementation in days. Cath Latr indicates whether microdialysis catheter was placed ipsilateral (Ipsi) or contralateral (Cont) to the cerebral hemisphere with greatest injury burden on CT. Patient GL-01 had two microdialysis catheters, perfused respectively with 4 and 8 mmol/L glucose at the same time. Patients GL-05 and GL-07 each had one catheter, and each catheter was perfused with 4 and 8 mmol/L glucose at different times with >24h between the two concentrations. All other patients had one microdialysis catheter each and one concentration of glucose (either 4 or 8 mmol/L). Failure of ISCUS*flex* analyser (but not microdialysis perfusion) for GL-02, GL-05, GL-07 and GL-16 during perfusion with 8 mmol/L glucose allowed NMR analysis of microdialysates but no ISCUS*flex* results. *Abbreviations: M, male; F, female; RTC, road traffic collision; ped, pedestrian; cycl, bicycle; GCS, Glasgow Coma Scale; ASDH acute subdural haemorrhage; ICH, intracerebral haemorrhage; EDH, extradural/epidural haemorrhage; DAI, diffuse axonal injury; ICP, intracranial pressure; CPP, cerebral perfusion pressure;* $PbtO_2$*, brain tissue oxygen tension; high resolution nuclear magnetic resonance analysis; NA, not available; TBI, traumatic brain injury; Ipsi, ipsilateral; Cont, contralateral*. Patients GL-17, GL-18, GL-19 and GL-20 were part of a previous study [11].

Supplemental Table 2. ISCUS*flex* clinical microdialysis analyser measurements.

ID	Age	Sex	Conc. Glucose Suppl. (mmol/L)	Unsupp. perfusion Glucose (mmol/L)	Suppl. perfusion Glucose (mmol/L)	Unsupp. perfusion Lactate (mmol/L)	Suppl. perfusion Lactate (mmol/L)	Unsupp. perfusion Pyruvate (µmol/L)	Suppl. perfusion Pyruvate (µmol/L)	Unsupp. perfusion LPR	Suppl perfusion LPR	Serum glucose† (mmol/L)	Serum lactate† (mmol/L)	Injury-Supp. Interval (hours)
**GL-01**	65	M	4	1.65	5.10	3.28	3.58	138	151	23.6	23.2	11.5	1.40	46
**GL-01**	65	M	8	0.73	8.97	1.85	2.07	66.8	69.7	27.7	29.6	11.5	1.40	46
**GL-03**	33	F	4	1.39	3.75	3.66	4.16	190	196	20.2	21.3	7.8	1.05	270
**GL-04**	37	M	4	2.74	3.77	3.07	3.71	106	119	28.9	31.2	7.8	0.95	66
**GL-05**	23	M	4	0.74	3.55	4.07	4.83	86.2	99	47.9	50	7.5	0.65	217
**GL-06**	19	F	4	0.79	4.56	2.31	1.79	69.8	52.8	31.2	31.9	7.9	1.10	57
**GL-07**	39	M	4	1.05	5.52	6.31	8.28	174	215	35.5	36.6	7.8	1.05	115
**GL-08**	44	M	8	0.81	8.31	2.39	2.16	80	65.9	30.1	32.9	7.6	1.25	47
**GL-09**	55	F	8	3.84	7.29	1.8	2.17	76.9	87.7	23.2	24.7	10.9	0.90	55
**GL-10**	41	F	4	0.42	2.90	4.18	5.2	118	130	35.8	39.9	6.6	0.65	138
**GL-11**	32	M	8	0.75	8.15	1.94	1.85	73.5	67.4	26.4	27.4	6.9	1.10	35
**GL-12**	16	F	8	3.82	6.39	2.57	2.11	166	146	15.5	14.5	6.1	0.70	41
**GL-13**	32	M	8	1.04	7.41	2.38	2.57	94.9	99.6	25.5	25.9	6.7	1.10	48
**GL-14**	32	M	8	1.23	6.39	5.57	4.73	146	138	39.5	34.3	9.6	1.45	67
**GL-15**	19	M	8	0.65	7.53	1.99	2.34	75.7	84.2	25.5	27.7	10.9	1.00	66
**GL-17**	27	M	4	0.42	3.24	2.85	4.48	99.8	191	21.4	22	6.6	NA	45
**GL-18**	53	F	4	1.91	4.52	1.05	1.29	52.7	74.4	17	17.4	5.7	NA	41
**GL-19**	37	M	4	1.45	4.28	2.12	2.19	99.1	99.1	21.3	22.1	6.9	NA	49
**GL-20**	28	M	4	0.83	3.60	2.92	3.31	109	122	23.1	26.4	8.3	NA	104
**mean**			**4**	**1.22***	**4.07**	**3.26***	**3.89**	**113***	**132**	**27.8**	**29.3**	**7.6**	**0.98**	**105**

**mean**	**8**	**1.61***	**7.56**	**2.56***	**2.50**	**98***	**95**	**26.7**	**27.1**	**8.8**	**1.11**	**51**

Table shows each patient's mean results, from baseline period (pre- and post- supplementation) and supplementation period (Supp.) respectively, with either 4 mmol/L or 8 mmol/L 1,2-$^{13}C_2$ glucose (via the microdialysis catheter) as indicated. Total number of patients whose data were included in this table was 18. One of these 18 patients (GL-01) had two catheters, so the results here are from 19 catheters. ***There was a statistically significant difference in baseline (unsupplemented) glucose, lactate and pyruvate between patients who received 4 mmol/L and 8 mmol/L 1,2-$^{13}C_2$ glucose (*p<0.001*, *lmer* in R). **†**Serum arterial glucose and lactate concentration shown was measured during the period in which 1,2-$^{13}C_2$ glucose was perfused via the cerebral microdialysis catheter. *Abbreviations: Conc., concentration; Suppl., supplemented; Unsupp., unsupplemented; LPR, lactate pyruvate ratio.* The bottom two rows show group means in bold type.

SUPPLEMENTAL METHODS

Supplemental details of acquisition of NMR spectra

^{1}H spectra were acquired using the water suppression pulse program zgpr, with 32 averages preceded by 2 dummy scans and a D1 (relaxation delay) of 32 seconds. ^{13}C spectra were acquired using the pulse program zgpg30, with 4,096 (4k) scans using a 30-degree flip angle on the carbon channel with a D1 of 3 seconds, digitizing 64k points. Power-gated broadband ^{1}H decoupling was achieved using the 'WALTZ-16' supercycle. The receiver gain was set to a constant value in each experiment. Analysis of spectra was performed after exponential filtering (0.3Hz for ^{1}H, 3.0Hz for ^{13}C), Fourier transformation, phase correction (automatic and manual) and automatic polynomial baseline correction, using TopSpin software (Bruker BioSpin GmbH).
